# KDIGO 2025 Clinical Practice Guideline for ADPKD: a commentary on intracranial aneurysms and other vascular manifestations from the ERA Working Group Genes & Kidney

**DOI:** 10.1093/ndt/gfag096

**Published:** 2026-05-01

**Authors:** Melanie M Y Chan, Tom Nijenhuis, Flavia Galletti, Lucile Figueres, Maria Vanessa Perez Gomez, Jan Halbritter, Roman-Ulrich Müller, Emilie Cornec-Le Gall

**Affiliations:** Medical Research Council Laboratory of Medical Sciences, Imperial College London, London, UK; Department of Nephrology, Imperial College Healthcare NHS Trust, London, UK; Department of Nephrology, Research Institute for Medical Innovations and Radboudumc Center of Expertise for Rare Kidney Diseases, Radboud University Medical Center, Nijmegen, The Netherlands; PKD International, Geneva, Switzerland; Service de Néphrologie et Immunologie Clinique, Centre de référence SORARE, Filière ORKID, Centre de référence Filière OSCAR, Nantes, France; Nantes Université, CHU Nantes, INSERM, Center for Research in Transplantation and Translational Immunology, UMR 1064, Nantes, F-44000, France; Department of Nephrology and Hypertension, IIS-Fundación Jiménez Díaz UAM, Madrid, Spain; Department of Medicine, RICORS2040, Madrid, Spain; Departamento de Medicina, Facultad de Medicina, Universidad Autónoma de Madrid, Madrid, Spain; Department of Nephrology and Medical Intensive Care, Charité Universitätsmedizin Berlin, Berlin, Germany; Renal Division, Faculty of Medicine, University Freiburg Medical Center, University of Freiburg, Freiburg, Germany; Department II of Internal Medicine, Faculty of Medicine and University Hospital, University of Cologne, Cologne, Germany; Center for Rare Diseases Cologne, Faculty of Medicine and University Hospital Cologne, University of Cologne, Cologne, Germany; Cologne Excellence Cluster on Cellular Stress Responses in Aging-Associated Diseases (CECAD), Cologne, Germany; Department of Nephrology, Medical Faculty, University Hospital Düsseldorf, Heinrich-Heine-University Düsseldorf, Düsseldorf, Germany; Service de Néphrologie, Hémodialyse et Transplantation Rénale, Centre de référence MARHEA, Filière ORKID, CHRU Brest, Brest, France; University Brest, Inserm, UMR 1078, GGB, Brest, France

**Keywords:** ADPKD, ICA, intracranial aneurysm, SAH, subarachnoid hemorrhage, thoracic aortic aneurysm

## Abstract

The 2025 KDIGO Clinical Practice Guideline for Autosomal Dominant Polycystic Kidney Disease (ADPKD) addresses intracranial aneurysms (ICAs) and other vascular manifestations, an aspect of ADPKD care which is of considerable clinical importance and associated with significant patient anxiety. This commentary from the ERA Working Group Genes & Kidney provides a European perspective on guideline implementation, highlighting key areas requiring action and research. People with ADPKD face a 4–5-fold increased risk of ICA (prevalence ∼12.9%) compared to the general population, yet absolute rupture rates remain low (0.57 per 1000 person-years). The guidelines appropriately emphasize risk-stratified, individualized approaches centered on family history, smoking history, and blood pressure control. Key points include the importance of patient information; selective screening in those with family history of ICA, subarachnoid hemorrhage, or unexplained sudden death; and use of contrast-free magnetic resonnance (MR) angiography as the preferred imaging modality. Rescreening intervals of 5–10 years are suggested for high-risk individuals with negative initial screening. Treatment decisions require multidisciplinary expertise at high-volume centers, balancing rupture risk against intervention complications. Thoracic aortic aneurysms show clear association with ADPKD, warranting family screening when present, while abdominal aortic aneurysms demonstrate no increased prevalence. Patient and clinician education emerges as paramount, moving beyond anxiety-driven approaches toward informed shared decision-making. Future research priorities include leveraging European registries to identify genetic risk modifiers, developing validated risk prediction tools, and establishing whether screening translates to improved clinical outcomes in ADPKD-specific populations.

## INTRODUCTION

Chapter 6 of the KDIGO 2025 Clinical Practice Guideline for Autosomal Dominant Polycystic Kidney Disease (ADPKD), dedicated to intracranial aneurysms (ICAs) and other vascular manifestations, addresses one of the most controversial and anxiety-inducing aspects of ADPKD management [1, 2]. Historical reports had already highlighted this vascular vulnerability. In 1885, Félix Lejars described fatal intracranial hemorrhages in adults with “gros reins polykystiques,” (big polycystic kidneys) already recognizing a link between ADPKD and cerebrovascular events [3]. More than a century later, this association is firmly established clinically, but the mechanisms underlying vascular susceptibility in ADPKD remain incompletely elucidated. Screening strategies for ICA in ADPKD have long fueled intense debate, often opposing an intuitive preventive stance to a more cautious, evidence-based approach [4–7]. The 2025 KDIGO ADPKD guidelines provide a carefully constructed and balanced perspective, offering much-needed elements for reflection in a field where clinical decisions are frequently influenced by perceived risk as much as by data.

The prevailing reflex among clinicians is straightforward: if aneurysm rupture can be prevented, why not screen everyone? Such reasoning, although understandably driven by a desire to “save lives,” risks oversimplifying a complex issue. Few events in medicine inspire greater dread than aneurysmal rupture, with its abruptness and devastating consequences. This intuition can obscure a more rational appraisal of actual risk and benefit.

Against this backdrop, the 2025 KDIGO ADPKD guideline refrains from dogmatic recommendations and instead encourages a nuanced, individualized approach. It acknowledges the tension between medical instinct and epidemiological reality, and emphasizes the focus toward informed decision-making, proportionality of care, and the central role of education. Continuous, structured education of both physicians and patients emerges as the key element to move beyond an overly cautious approach, toward a more rational, data-driven, and patient-centered management of vascular risk in ADPKD. The purpose of this article, on behalf of the ERA Working Group Genes & Kidney, is to provide an overview of the latest and most impactful aspects for the field, with specific reference to the European context. This commentary serves to support implementation of the guidelines, to highlight where action is needed in clinical care and to identify areas with an urgent need for research.

## THE IMPORTANCE OF PROVIDING ADEQUATE INFORMATION TO PATIENTS AND FAMILIES

The 2025 KDIGO guidance represents an important shift toward empowering people with ADPKD through education and risk awareness. We welcome this direction where risk communication around ICAs has largely been inconsistent and poorly implemented [8]. While subarachnoid hemorrhage in people with ADPKD is a rare event (0.57 per 1000 person-years; 95% confidence interval 0.19–1.14, which corresponds pragmatically to approximately one rupture every 6 years in a large center following 300 ADPKD patients), given its potentially devastating consequences, it is understandably one of the main concerns highlighted by patients and health professionals [9]. From a European perspective, where healthcare systems prioritize shared decision-making and patient autonomy, we fully support the recommendation to provide patients with comprehensive information about their risk of ICA. We have included a dedicated patient information sheet that can be used to facilitate discussions around ICA risk (Appendix 1 in the supplemental materials).

People with ADPKD have a 4–5-fold increased risk of developing an ICA with an estimated 12.9% (95% CI 10.4–15.4) prevalence in the ADPKD population compared to 2.9% (95% CI 1.9–4.5) in the general population [1, 10] (Fig. 1). In those with a positive family history, the prevalence of ICA rises to 17.1% (95% CI 13.4–21.1) and as seen in the general population, women, particularly post-menopause, have an increased risk of ICA and SAH [5, 11]. Although consistent in direction, estimates for unruptured ICAs exhibit significant heterogeneity, influenced by varying screening protocols across center and referral and ascertainment biases. This variability underscores the need for large, harmonized datasets, and future studies should harness the power of large ADPKD data registries, such as the European Rare Kidney Disease Registry (ERKReg) and the UK’s National Registry of Rare Kidney Diseases, to establish more precise estimates of ICA prevalence and construct prognostic tools to better predict risk of rupture in this population.

**Figure 1:**
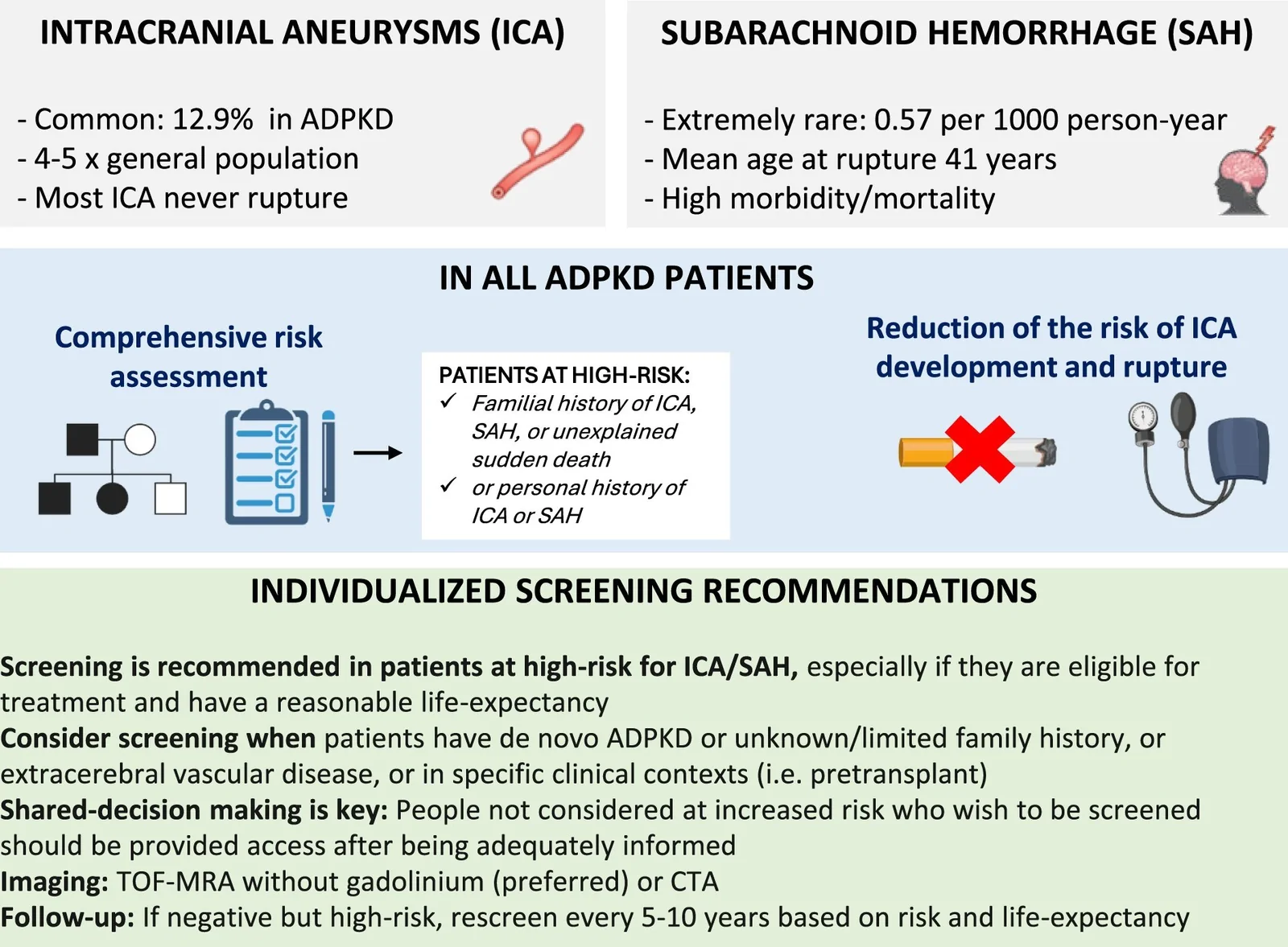
Epidemiology, risk stratification and recommendations for screening of intracranial aneurysms in ADPKD. Abbreviations: ADPKD: autosomal dominant polycystic kidney disease, CTA: computed tomography angiography, ICA: intracranial aneurysms, SAH: subarachnoid hemorrhage, TOF: time-of-flight.

Box 1. **ICA, SAH and information for patients.**

*Recommendation 6.1.1: We recommend informing adults with ADPKD about the increased risk for intracranial aneurysms (ICAs) and subarachnoid hemorrhage (1C)*.

G&K comment: Although transparent communication regarding this heightened risk may induce anxiety and influence life decisions, we recognize that it empowers patients and families to make well-informed choices, particularly regarding screening and modifiable risk factors. It is also crucial to clarify that most ICA do not rupture. In Europe, where literacy levels and access to digital tools vary, we recommend that education be culturally sensitive, accessible in multiple languages, and ideally delivered verbally accompanied by written or visual aids. We provide a patient information sheet (Appendix 1 in the supplemental materials), co-produced by G&K and a patient representative, to facilitate these discussions.

*Practice Point 6.1.1: All people with ADPKD should be educated to recognize thunderclap headache, characterized by a severe sudden-onset headache that reaches its maximum intensity within seconds to a minute. Recognition of such symptoms should prompt immediate medical attention*.

G&K comment: Given the significant morbidity and >30% mortality [12] associated with ruptured ICAs, we strongly support this potentially lifesaving recommendation but acknowledge such information is likely to induce anxiety in some people with ADPKD and should therefore be presented carefully, with appropriate reassurance. Given that the median age at aneurysm rupture is ∼10 years earlier than in the general population (41 years versus 52 years) [11, 13], we suggest that emergency departments establish a low threshold for imaging, even in young individuals with ADPKD. Simple measures such as emergency information cards—paper or digital—can also facilitate timely recognition and management.

*Practice Point 6.1.2: A detailed personal history of SAH and a family history of ICA, SAH, and unexplained sudden death should be obtained to identify people with ADPKD who are at higher risk for ICA*.

G&K comment: A comprehensive family history, spanning at least three generations, serves as a cost-effective and highly effective clinical tool for stratifying the personal risk of both kidney failure and ICA. We recommend that this is revisited on a regular basis [14]. A positive family history increases the risk of ICA by approximately four-fold in the ADPKD population, comparable to the general population [15]. Common genomic variants account for 21%–29% of the heritability of ICAs in the non-ADPKD population, supporting a polygenic basis for familial clustering of ICAs [16]. Future research in ADPKD cohorts is needed to explore the potential of ICA genomic risk scores in personalized screening strategies. Despite the clear familial association, it is however important to acknowledge that ∼50%–60% of individuals diagnosed with an ICA and ∼40% with a ruptured ICA do not have a family history [5, 11].

*Practice Point 6.1.3: Because smoking is a strong modifiable factor for ICA development and rupture, healthcare providers should ask all people with ADPKD about their tobacco use, advise them to stop using tobacco, and provide behavioral interventions and approved pharmacotherapy for cessation, if needed*.

*Practice Point 6.1.4: Because uncontrolled hypertension is a moderate modifiable factor for ICA development and rupture, early diagnosis and adequate treatment of hypertension is indicated in people at risk of or diagnosed with ADPKD, particularly those at an increased risk for ICA*.

G&K comment: Smoking (especially >20 pack-years) and hypertension are modifiable risk factors which are independently associated with a two-fold increased risk of ICA [11]. We strongly support these recommendations which align with broader public health strategies to reduce cardiovascular disease and acknowledge that smoking cessation is often under-addressed in nephrology. Indeed, Europe has the second highest prevalence of tobacco smoking among adults globally (25%; World Health Organisation 2025) with rates that vary from 6.4% in Sweden to 28.7% in Bulgaria (https://ec.europa.eu/eurostat/statistics-explained/index.php?title=Tobacco_consumption_statistics) highlighting the need for region-specific smoking cessation initiatives.

Similarly, early and sustained blood pressure control is essential to slow ADPKD progression and reduce the risk of ICA development and rupture. In line with KDIGO guidance, a systolic blood pressure ≤120 mmHg should be targeted through a combination of lifestyle modifications and renin-angiotensin system inhibition with a lower target of ≤110/75 mmHg for those 18–49 years with an eGFR > 60 ml/min/1.73m^2^ [2]. Home blood pressure monitoring through digital apps and pharmacist-led interventions could be used to enhance adherence and detection of uncontrolled hypertension in ADPKD. Other modifiable risk factors such as low physical activity and excessive alcohol intake have also been linked to an increased risk of SAH in the general population and their relevance to people with ADPKD should be explored [17, 18].

*Practice Point 6.1.5: People with ADPKD should be informed of the implications of ICA screening*.

G&K comment: Informing patients about the risks and benefits of ICA screening requires a nuanced discussion and adequate time. Patients should understand both the potential for early detection and the risks of overdiagnosis, false reassurance, and anxiety (Fig. 1). Given the complexities involved, there is a need to create tailored medical education tools to effectively convey these risks and facilitate collaborative decision-making (Appendix 1 in the supplemental materials).

## SCREENING: IN WHOM, WHEN, HOW OFTEN?

Defining the indications of screening for diseases or disease manifestations such as aneurysms in ADPKD is a delicate process which must consider several key aspects (Fig. 1). (1) Disease burden and prevalence, (2) availability of accurate testing procedures, (3) proven benefit of early treatment, (4) safety of both testing and treatment procedures, and (5) socioeconomic considerations [19]. Both physicians and patients frequently assume intuitively that screening is beneficial. However, the situation gets more complex when really considering all five points. Applied to ICA screening in ADPKD, these principles lead to the following considerations. The burden of a ruptured aneurysm is very high. However, the majority of aneurysms detected are small (inferior to 5 mm) and localized in the anterior circulation, indicating a low rupture risk *per se* [5, 20]. The prevalence and incidence of aneurysms in ADPKD as well as their rupture risk depends on patient-specific characteristics with a positive family history for (ruptured) aneurysms being the central point [11]. This underlines the importance of obtaining a thorough family history of genetic kidney disease, not limited to renal manifestations [14, 20]. Safety of the testing approach is very highly based on the standard of contrast-free magnetic resonance imaging with time-of-flight (TOF) angiography. The importance of treatment of an aneurysm with a high rupture risk is undisputed. However, the intervention itself comes with considerable complications. A meta-analysis of over 100 000 individuals from the general population reported a 30-day clinical complication risk of ∼5% and a fatality risk of 0.3% for endovascular treatment, while for neurosurgical treatment the pooled clinical complication risk was 8.3% and the fatality risk 0.1% [21]. Importantly, two studies reported higher complication rates associated with cerebral angiography or treatment of unruptured ICAs in people with ADPKD compared with those without ADPKD, although these findings must be interpreted in light of older treatment eras and evolving interventional techniques [22, 23]. The detection of a small aneurysm which does not reach the threshold for intervention can place significant psychological strain on the patient. Conversely, such detection may carry a positive impact by reinforcing patient engagement in smoking cessation, blood pressure control, and broader cardiovascular risk reduction. Besides, screening does not preclude the de novo formation of aneurysms after the test. The socio-economic impact of aneurysm screening has not been entirely ascertained and has been a matter of active debate. The combination of all factors raised above as well as considerable remaining uncertainty regarding aneurysm epidemiology due to study design is the basis for an ongoing active debate on the role of aneurysm screening in ADPKD [4, 6, 7]. Consequently, and in line with the currently available data foundation, the KDIGO guideline has based its recommendations on the assumption that potential harms, in certain situations, could outweigh the benefits of general screening and chosen a tailored, personalized approach (Fig. 2). Nonetheless, and importantly, there is specific emphasis on patient information (see Supplementary material) leaving room for an individualized decision-making process based on patient preference. The guideline sets the stage with clear recommendations on who should be screened considering individual risk, defines screening intervals (practice point 6.1.11) and test modalities (practice point 6.1.10), but also leaves room for well-informed patients to make up their minds in the context of a shared decision-making process. Considering the lack of data and study-design limitations, this results in one recommendation (6.1.2, graded 1D) and six practice points, which provide guidance in specific clinical settings such as limited family history (6.1.6), kidney transplantation (6.1.7), personal preference (6.1.8.), and pregnancy (6.1.9).

**Figure 2:**
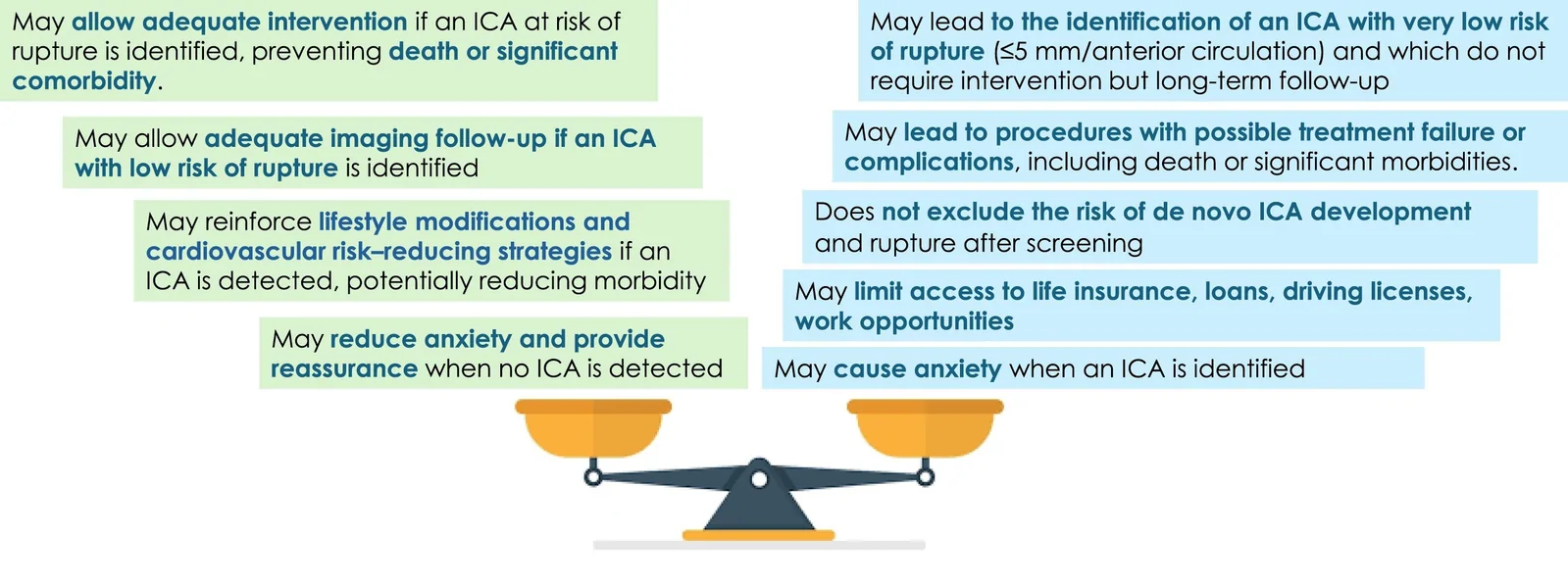
Advantages and disadvantages of screening for intracranial aneurysms in ADPKD.

Box 2. **Screening for ICA in ADPKD**

*Recommendation 6.1.2: We recommend screening for ICA in people with ADPKD and a personal history of SAH or a positive family history of ICA, SAH, or unexplained sudden death in those eligible for treatment and who have a reasonable life expectancy (1D)*.

G&K comment: A selective approach to ICA screening in people with ADPKD is reasonable and the only non-modifiable risk factor supported by sufficient data is family history. Screening should begin at age >18 years in at-risk individuals, as ICA rupture is exceedingly rare in children, but should not be delayed too long after 18–20 years given the risk of aneurysm development in young adults. Life expectancy needs to be considered in all screening programs. High-risk occupations in which a sudden rupture may compromise others (e.g. pilots) may be a potential reason to consider screening beyond this recommendation and may be required by local legislation. This can also be the case in specific medical situations (see Practice Point 6.1.7).

*Practice Point 6.1.6: Screening for unruptured ICA also should be discussed for people with de novo ADPKD, those with unknown familial history or a small number of ADPKD-affected relatives, and those with personal or familial history of extracerebral vascular phenotype*.

G&K comment: As the basis to selection for screening is family history, this practice point opens screening strategies to patients with limited or negative family history. Extracerebral vascular manifestations may point toward a potential general weakness of blood vessel walls, a reasonable assumption, which, however, is based on pathophysiological assumptions rather than a broad body of data.

*Practice Point 6.1.7: Screening for unruptured ICA also can be discussed in specific clinical settings, such as in the context of evaluation for kidney and/or liver transplantation or before major elective surgery*.

G&K comment: We agree that specific clinical settings warrant consideration of ICA screening. Transplant centers currently handle this point differently, but screening in the context of transplantation evaluation is reasonable given the potential consequences for the recipient. In the context of living donation, such evaluation may also inform the overall transplant strategy (e.g. timing, need for prior aneurysm management), thereby also impacting the donor. A targeted decision on aneurysm management may be particularly helpful for wait-listed patients.

In contrast, there is currently no supporting data to justify ICA screening before major elective surgery. While this practice is implemented in some centers, the absence of evidence demonstrating benefit does not support its routine consideration. If one accepts “major elective surgery” as a trigger to discuss screening, one could theoretically ask whether labor and delivery should be treated analogously. Population-based data, however, do not show an increased risk of aneurysm rupture during pregnancy or delivery.

*Practice Point 6.1.8: People with ADPKD who are not considered at increased risk for ICA and who, after comprehensive information, prefer being screened for ICA should be given access to screening*.

G&K comment: Shared-decision making in the context of accurate risk communication and comprehensive information is very important considering the devastating consequences of aneurysm rupture. We do however acknowledge that access to screening varies between countries and may impact implementation of this practice point.

*Practice Point 6.1.9: In women with ADPKD and either a family history of ICA, SAH, or unexplained sudden death; de novo ADPKD; unknown familial history; or a small number of ADPKD-affected relatives, screening for unruptured ICA should precede pregnancy planning*

G&K comment: In women with ADPKD who are considered at increased risk for ICA—including those with a family history of ICA, SAH, or unexplained sudden death; de novo ADPKD; unknown familial history; or a small number of ADPKD-affected relatives—screening for unruptured ICA should be performed and, when possible, discussed and completed prior to pregnancy planning. This approach aims to ensure optimal timing of potential management decisions and to avoid the need for screening during pregnancy, when diagnostic and therapeutic options are more limited. It is also relevant to note, considering that this question occurs frequently, that there is no evidence-based justification for discouraging vaginal delivery in women with ADPKD, neither based on renal nor (potential) extrarenal manifestations including the risk of ICA.

*Practice Point 6.1.10: Time-of-flight magnetic resonance angiography (MRA) without gadolinium enhancement should be the method of imaging when screening is to be pursued for ICA in people with ADPKD. High-resolution computed tomography angiography (CTA) can be used as an alternative*.

G&K comment: TOF-MRA without contrast agents provides high sensitivity and specificity for intracranial aneurysms and comes without the risk associated with radiation and/or contrast exposure, the rational for this practice point is therefore very reasonable. Of note, 1.5 Tesla (T) TOF-MRA may occasionally overcall small vascular irregularities, leading to false-positive suspicion of ICA. Therefore, any uncertain or borderline finding should be evaluated by a neuroradiologist, who can determine whether repeat imaging at 3T is warranted to refine characterization and avoid unnecessary patient anxiety.

*Practice Point 6.1.11: If the screening is negative in people with a high risk of ICA, timing of rescreening should be individualized, possibly every 5–10 years, based on risk factors, age, and life expectancy*.

G&K comment: Considering the increased risk of multiple aneurysms and de novo aneurysm formation in patients with ADPKD and a high risk based on personal and/or family history, repeat screening is warranted with intervals based on patient characteristics as well as preference. Although the practice point mentions a range of 5–10 years, it is important to note that most non-ADPKD familial aneurysm screening programs (e.g. in families with ≥2 first-degree relatives with ICA or SAH) have used repeat imaging roughly every 5 years, and cost-effectiveness models suggest intervals of 5–7 years in high-risk groups [24]. Major US and European guidelines (AHA/ASA, ESO) recommend screening in individuals with strong familial risk but do not provide a formal rescreening interval, underscoring that this remains an expert-opinion area where a shorter interval may be appropriate when additional risk factors are present (e.g. poorly controlled hypertension, smoking, or multiple affected relatives). If screening is performed not due to a high-risk situation but personal preference in the first place, repeat imaging is usually not recommended. However, this will, again, require a shared decision-making process.

## TREATMENT OF ICAS IN ADPKD

The KDIGO guideline stresses the fact that only limited information is available on the natural history of asymptomatic ICAs in ADPKD, nor are there predictive tools, but the risk of rapid expansion or rupture of small ICAs detected by screening appears to be low. Evidence is largely inferred from experience from ICAs in other non-ADPKD populations and with respect to treatment of ICAs, the guideline therefore refers to the 2022 European Stroke Organisation guidelines on management of unruptured ICAs [25]. This latter guideline advises to escalate to preventive aneurysm repair if the estimated 5-year risk of aneurysm rupture, for which little data is available in the ADPKD population, is higher than the risk of the preventive treatment modality. In cases where ICAs have been shown to grow on repeat imaging, preventive treatment is recommended but still needs to be weighed against risk of treatment. Thus, decisions regarding the management of ICAs and, if indicated, the choice of treatment modality should be assessed in a multidisciplinary setting (neurovascular boards) including experienced diagnostic radiologists, neurosurgeons, and neurointerventional radiologists. When treatment is not indicated, radiologic monitoring to detect ICA growth, morphologic modification, and de novo ICAs should be continued at individualized intervals as long as preventive treatment remains an option.

Box 3. **Treatment of ICAs in ADPKD**

*Practice Point 6.1.12: When one or several ICAs are identified, treatment options, such as conservative management and microvascular or endovascular repair, should be assessed within a multidisciplinary setting at centers of expertise with high ICA case volumes*.

G&K comment: We subscribe to the above practice point and emphasize the importance of a multidisciplinary approach based on centers of expertise for ICA management. Also in this decision-making process, given the limited evidence especially in the ADPKD population but also to a lesser extent in the non-ADPKD population, this calls for shared decision making between the expert physician and the patient, based on the recommendation by the multidisciplinary team and patient-related psycho-social factors and preferences.

### OTHER VASCULAR MANIFESTATIONS IN ADPKD

Beyond ICAs, patients with ADPKD may develop various cardiovascular complications, including carotid, coronary, and vertebral artery dissections, and thoracic aortic aneurysms. The determinants of these manifestations are unknown, as is whether pathogenic mechanisms overlap with those underlying ICAs, despite differing arterial wall structures. A prospective vascular complications registry would advance understanding of prevalence and risk factors.

Importantly, abdominal aortic aneurysms (AAA) show no increased prevalence in ADPKD, whereas thoracic aortic aneurysms (TAA) demonstrate clear association, particularly with hypertension [26–28]. This distinction has screening implications and should clarify a common misconception equating AAA and TAA risk in ADPKD.

Lastly, the 2025 KDIGO ADPKD guideline does not discuss whether ADPKD predisposes to increased post-transplant vascular complications—arterial thrombosis, dissection, anastomotic complications, or impaired arteriovenous fistula development—and this merits systematic investigation. Experimental evidence demonstrates that endothelial-specific deletion of *Pkd1* in mice impairs arteriovenous fistula maturation and promotes endothelial dysfunction, suggesting that altered vascular biology in ADPKD may affect surgical vascular outcome [22]. Longitudinal observational studies in ADPKD patients undergoing vascular procedures would be valuable to determine whether this translates to clinically relevant differences in outcomes.

Box 4. **Other vascular phenotypes in ADPKD.**

*Practice Point 6.2.1: Routine screening of vascular abnormalities of non-intracranial large arteries has no role in people with ADPKD and no familial history of vascular aneurysms or dissections*.

G&K comment: We agree with this practice point and emphasize that current data on extracranial vascular abnormalities in ADPKD (coronary, cervicocephalic, vertebral, thoracic aorta) are largely limited to case reports and small series. Routine imaging of large non-intracranial arteries in unselected ADPKD patients is therefore not justified. Importantly, an adult with ADPKD and CKD remains a CKD patient at high risk of major adverse cardiovascular events, and standard cardiovascular work-up and prevention according to general CKD and cardiovascular guidelines should not be overlooked.

*Practice Point 6.2.2: People with ADPKD and their first-degree relatives who have a family history of aortic root or thoracic aortic aneurysms should be screened for aortic aneurysms*.

G&K comment: We support this practice point. Although aortic root and thoracic aortic aneurysms are very rare in ADPKD, family screening in the presence of a positive family history is often overlooked. Increasing clinician awareness and discussing such extracranial vascular findings with ADPKD reference centers can improve appropriate cascade screening of first-degree relatives.

*Practice Point 6.2.3: In people with ADPKD and dilatation of the aortic root or thoracic aortic aneurysm, therapeutic measures to limit aortic expansion should be offered; these include smoking cessation, statin therapy, and antihypertensive therapy including a beta-blocker and an ACEi or ARB*.

G&K comment: The rationale for beta-blockers in thoracic aortic aneurysms derives primarily from murine models, Marfan syndrome data, and observational studies in Marfan patients. Recent meta-analyses of randomized controlled trials (albeit limited to 161 patients across 4 RCTs) found no statistically significant benefit from beta-blocker treatment in thoracic aortic aneurysms [29]. Current guidelines recommend beta-blockers as reasonable for blood pressure control (Class IIa) in sporadic thoracic aneurysms when BP ≥ 130/80 mmHg, with stronger evidence (Class I) only in Marfan syndrome [30]. Whether Marfan-derived evidence generalizes to non-Marfan populations (including ADPKD) remains uncertain, but this approach is reasonable in the absence of specific data for ADPKD-associated aneurysms.

*Practice Point 6.3.1: Echocardiography at baseline with occasional repeat echocardiograms should be offered in people with ADPKD who have a history of severe or uncontrolled hypertension, a heart murmur, signs or symptoms of cardiac dysfunction, other cardiovascular manifestations, or a familial history of thoracic aortic aneurysm (TAA) or nonischemic cardiomyopathy*.

G&K comment: Given that many ADPKD patients have hypertension at diagnosis, these criteria may apply to a substantial proportion of patients, making consistent implementation and clinician awareness crucial. Studies on implementation and actionability would be valuable. Two single-center studies highlight potential cardiac involvement: one reported high prevalence of left ventricular hypertrophy and valvular abnormalities in largely asymptomatic ADPKD patients, while a large case series identified congenital heart disease in 1.84% of ADPKD patients, higher than the general population [31, 32]. Most valvular disease patients are asymptomatic and may lack audible murmurs, emphasizing that cardiac manifestations may be under-recognized in asymptomatic individuals.

### CONCLUSION AND PERSPECTIVE

Patient and professional education emerges as the cornerstone of ICA management in ADPKD. This is a field that demands careful guidance to prevent patients from living under a sword of Damocles while appreciating that absolute risks remain very low—approximately one rupture per 1800 patient-years. Despite advances in imaging and multidisciplinary care, we still lack a fundamental understanding of what determines ICA formation in ADPKD. International studies to identify genetic risk factors are ongoing, though the complex polygenic architecture and relatively modest sample sizes pose significant challenges. Nevertheless, large-scale collaborations leveraging registries will eventually succeed in identifying risk modifiers, paving the way for precision medicine and more targeted screening strategies. Critically, ICA screening must not overshadow other preventive measures—particularly cardiovascular disease surveillance and age-appropriate cancer screening––which are likely to have a broader impact on overall mortality in the ADPKD population. Moving forward, European collaboration through harmonized databases and prospective registries represents our best opportunity to better understand ICA determinants, refine risk stratification, optimize screening intervals, and ultimately deliver truly personalized care that balances vigilance with quality of life for people living with ADPKD.

## Supplementary Material

gfag096_Supplemental_File

## Data Availability

No original data was involved in the creation of this manuscript.
