# Supplementary material for: KDIGO 2025 Clinical Practice Guideline for ADPKD: a commentary on intracranial aneurysms and other vascular manifestations from the ERA Working Group Genes & Kidney
Source: Nephrol Dial Transplant. 2026 May 1;41(11):2083–90. doi: 10.1093/ndt/gfag096 (PMC13719708; doi:10.1093/ndt/gfag096)
Supplement: Supplementary file 1 [file gfag096_supplemental_file.docx]

**Annex 1 – Patient information sheet**

**Brain Aneurysms and ADPKD**

*Understanding your risk and screening options*

**What is a brain aneurysm?**

A brain (intracranial) aneurysm is a small "bulge" or weak spot in the wall of a blood vessel in the brain.

Because the wall of the blood vessel is weaker at that spot, there is a small chance it could tear or burst (called a rupture). If this happens, blood leaks into the space around the brain, which is a medical emergency and can be life-threatening. However, this is very uncommon

**Key point** : most aneurysms stay small, remain stable over time, and never cause symptoms.

**Why do we talk about brain aneurysms in ADPKD?**

People with polycystic kidney disease (ADPKD) are more likely to develop a brain aneurysm than people without ADPKD, although the exact mechanisms remain unclear.

**How common are aneurysms and ruptures?**

- In the general population, about 3 in every 100 people have an aneurysm
- In people with ADPKD, about 13 in every 100 adults have one.

**Ruptures** are very rare. Large studies show that less than one aneurysm ruptures per 1,000 people each year.

**Out of 2,000 people with ADPKD:**

- **1,740** have no aneurysm
- **260** have an intracranial aneurysm (ICA)
- **~1** would experience a rupture in a given year

**What this means:** Among 2,000 adults with ADPKD, screening would detect about 260 with an aneurysm. However, only about 1 rupture would occur per year in this entire group. Most people with ADPKD will never experience a rupture. Even if an aneurysm is found, the chance of it bursting is very low.

**Key point:** Most aneurysms never burst (rupture) and it is very rare for this to happen.

**Who is at higher risk?**

Certain factors increase the risk of aneurysm or rupture:

- A first-degree relative (mother, father, brother, sister, children) with an aneurysm, brain bleed, or sudden unexplained death
- If you have had an aneurysm or a brain bleed before
- Active smoking
- Uncontrolled high blood pressure

**Good news:** The last two factors can be changed! Quitting smoking and keeping your blood pressure under control can significantly lower your risk of an aneurysm or rupture.

**Who should be screened for intracranial aneurysms?**

Aneurysms can be found with MRI scans of the head. MRI screening is not recommended for everyone. It is recommended or strongly considered for:

- People who have had an aneurysm or bleeding in the brain before
- People with a close family member (parent, sibling, or child) who had an aneurysm, brain bleed, or died suddenly without explanation.

Screening may also be discussed in these situations:

- De novo ADPKD (meaning neither parent had ADPKD)
- Families with only a few people affected by ADPKD, because predicting risk may be harder
- Before a kidney or liver transplant, according to transplant center practice
- For adults who choose screening after getting clear information

**Shared Decision-Making:** Choosing to be screened is a personal decision. Discuss the benefits and limitations with your doctor based on your specific situation.

**Screening: weighing up the benefits and limitations**

**Benefits**

- If a high-risk aneurysm is found, treatment may be offered to prevent rupture
- If a low-risk aneurysm is found, your medical team can plan safe follow-up.
- A normal result can be reassuring.

**Limitations**

- Small aneurysms may be found that would never cause a problem but still need long-term monitoring.
- Follow-up scans can cause worry for some people
- Treatment carries risks, even with less invasive procedures
- MRI is not comfortable for everyone and access may vary depending on where you live.
- Screening does not remove all risk—aneurysms can develop after screening.
- Other findings may appear on the scan which could affect things like insurance, loans, specific occupations or driving licenses in some countries.

**What happens if an aneurysm is found?**

If a higher-risk aneurysm requires treatment, it is most often managed with an endovascular approach—a thin tube (catheter) inserted through an artery to treat the aneurysm from the inside. Open surgery is now uncommon. Decisions are made by a specialist team to ensure the safest option for you.

**Important:** If an aneurysm is found, tell close relatives with ADPKD so they can talk about screening with their doctor.

**What can I do to reduce my risk?**

- Don't smoke (or ask for help to quit if you do)
- Keep your blood pressure well controlled
- Attend regular follow-up appointments if recommended
- Share your family history with your care team

**What about screening after transplant?**

If you've had a kidney or liver transplant, the aneurysm risk doesn't go away. If you were considered at risk before transplant, your doctor may recommend continuing follow-up screening after transplantation if you have good overall health and no major conditions that would prevent treatment of an aneurysm if one is found.

In other words: screening makes sense if treatment would be an option for you. Talk with your transplant team to decide if ongoing monitoring is right for your situation.

**Warning signs: when to seek emergency care**

**Signs of aneurysm rupture**

A rupture typically causes sudden, severe symptoms:

- **Sudden, extremely severe headache** ("thunderclap headache")
- Neck stiffness
- Feeling sick or vomiting
- Difficulty speaking or moving
- Loss of consciousness

**👉 Call emergency services immediately and tell them you have ADPKD.**

**Key takeaways**

- ADPKD increases the risk of brain aneurysms, but most aneurysms stay stable and never cause problems.
- Ruptures are extremely rare.
- Screening is mainly recommended if you have a personal or family history of aneurysm or brain bleed.
- If an aneurysm is found, a specialist team will weigh the benefits and risks of treatment versus monitoring.
- You can lower your risk: quit smoking and keep your blood pressure under control.
- Screening is your choice—talk with your doctor to decide that is best for you.

*This information sheet was developed for patients with ADPKD. For questions about your individual situation, consult your healthcare team.*
